# A randomized double-blind, placebo-controlled trial to evaluate the safety and efficacy of live *Bifidobacterium longum* CECT 7347 (ES1) and heat-treated *Bifidobacterium longum* CECT 7347 (HT-ES1) in participants with diarrhea-predominant irritable bowel syndrome

**DOI:** 10.1080/19490976.2024.2338322

**Published:** 2024-04-17

**Authors:** S Srivastava, U Basak, M Naghibi, V Vijayakumar, R Parihar, J Patel, PS Jadon, A Pandit, RR Dargad, S Khanna, S Kumar, R Day

**Affiliations:** a Clinical Development & Science Communications, Vedic Lifesciences Pvt Ltd, Mumbai, India; b Medical Department, ADM Health & Wellness, London, UK; c Gastroenterology Department, Gastroplus Digestive Disease Centre, Ahmedabad, India; d Gastroenterology Department, Apex Gastro Clinic and Hospital, Ahmedabad, India; e Medicine Department, Jaipur National University Institute for Medical Science & Research Centre, Jaipur, India; f General Surgery Department, United Multispeciality Hospital, Maharashtra, India; g Medicine Department, Lilavati Hospital & Research Centre, Maharashtra, India; h Gastroenterology Department, Criticare Asia Multispeciality hospital, Maharashtra, India; i Independent Biostatistical Consultant, Delhi, India

**Keywords:** IBS-D, probiotic, postbiotic, Gut microbiome, IBS-SSS, abdominal pain, QoL

## Abstract

To determine the efficacy of the probiotic *Bifidobacterium longum* CECT 7347 (ES1) and postbiotic heat-treated *Bifidobacterium longum* CECT 7347 (HT-ES1) in improving symptom severity in adults with diarrhea-predominant irritable bowel syndrome (IBS-D), a randomised, double-blind, placebo-controlled trial with 200 participants split into three groups was carried out. Two capsules of either ES1, HT-ES1 or placebo were administered orally, once daily, for 84 days (12 weeks). The primary outcome was change in total IBS-Symptom Severity Scale (IBS-SSS) score from baseline, compared to placebo. Secondary outcome measures were stool consistency, quality of life, abdominal pain severity and anxiety scores. Safety parameters and adverse events were also monitored. The change in IBS-SSS scores from baseline compared to placebo, reached significance in the ES1 and HT-ES1 group, on Days 28, 56 and 84. The decrease in mean IBS-SSS score from baseline to Day 84 was: ES1 (−173.70 [±75.60]) vs placebo (−60.44 [±65.5]) (*p* < .0001) and HT-ES1 (−177.60 [±79.32]) vs placebo (−60.44 [±65.5]) (*p* < .0001). Secondary outcomes included changes in IBS-QoL, APS-NRS, stool consistency and STAI-S and STAI-T scores, with changes from baseline to Day 84 being significant in ES1 and HT-ES1 groups, compared to the placebo group. Both ES1 and HT-ES1 were effective in reducing IBS-D symptom severity, as evaluated by measures such as IBS-SSS, IBS-QoL, APS-NRS, stool consistency, and STAI, in comparison to the placebo. These results are both statistically significant and clinically meaningful, representing, to the best of the authors’ knowledge, the first positive results observed for either a probiotic or postbiotic from the same strain, in this particular population.

## KEY MESSAGES

What is already known on this topic

IBS is a chronic functional gastrointestinal disorder characterized by abdominal pain, bloating and abnormalities in stool frequency or form. The gut microbiota of people living with IBS differs markedly to the microbiota of healthy individuals. Gut microbiota may play a key role in IBS aetiology and IBS symptoms may be alleviated by modulating the gut microbiota. Several proposed ways to modulate gut health include normalizing the gut microbiota, preventing the overgrowth of pathogenic bacteria, modulating visceral afferent pathways, and enhancing intestinal barrier function. However, significant heterogeneity between studies, study quality and population, study design and concerns about sample size have limited national and supranational bodies from recommending probiotics for IBS. Further well-powered, randomized, repeatable and controlled trials are warranted.

What this study adds

The results of this study substantially contribute to the IBS research field, firstly by providing clinically meaningful and statistically significant results from a rigorous, well designed randomized, placebo-controlled trial and secondly, by exploring the use of postbiotics in IBS, an area of research still in its infancy. Probiotic (ES1) and postbiotic (HT-ES1) supplementation significantly reduced IBS symptom severity scores compared to placebo. This study met primary and secondary outcomes and strongly suggest that ES1 and HT-ES1 could be beneficial in the management of IBS.

How this study might affect research, practice, or policy

This study adds to the current evidence base, supporting the use of probiotic/postbiotics for IBS. This research could be used to inform health professionals about using probiotics in IBS and help improve the quality of life and wellbeing for people living with the condition.

## 1. Introduction

Irritable bowel syndrome (IBS) is a functional gastrointestinal disorder characterized by abdominal pain, fecal abnormalities and altered bowel habits.^1^ Symptoms can be debilitating and significantly impact quality of life (QoL).^2^ IBS is a multifactorial disorder, and its complex pathophysiology is still not fully understood. Functional abnormalities, such as altered visceral sensitivity, bowel motility and secretory dysfunctions alongside structural abnormalities, such as microbial imbalances, are contributory factors underlying IBS pathophysiology.^3^

The global prevalence of IBS is estimated to be 11.2%, with rates of 5–15% reported for most European countries, the United States and China.^4^ The most recent global study conducted by the ROME foundation across 33 countries reported IBS rates between 3% and 5%.^5^

IBS is a diagnosis of exclusion based on patient symptoms and is classified by the predominant bowel habits; diarrhea-predominant IBS (IBS-D), constipation-predominant IBS (IBS-C) and mixed bowel habits (IBS-M).^6^ Management depends on the main presentations and may include pharmacological intervention such as antispasmodics, antidiarrheals and laxatives, diet modification and psychotherapy.^7^ However, given the multifactorial nature of IBS, there is currently no single management strategy for a particular subtype that has been universally adopted.^8^ Recent studies suggest agents such as probiotics, that modulate the gut microbiota, may help manage IBS.

Probiotics are defined as live microorganisms that, when administered in adequate amounts, confer a health benefit on the host.^9^ Several probiotics, particularly of the *Lactobacilli* and *Bifidobacterium* genera, have been suggested for the management of IBS.^10^ Several systematic reviews have been published on using probiotics to manage IBS.^11–13^ A recent review concluded that for the IBS-D subtype, probiotics may be an effective solution for improving abdominal pain and distension,^11^ Zhang et al. (2016) reported that single-strain, low-dose probiotics appeared to be more successful in improving symptom response and QoL,^12^ and Konstantis et al. (2023) demonstrated a positive effect on pain and bloating.^13^ A recently published meta-analysis compared conventional treatment and probiotic interventions in participants with IBS, and results indicated that both interventions were efficacious in improving persistence of IBS symptoms and abdominal pain scores. The meta-analysis concluded that larger RCTs focusing on specific IBS subtypes and specific probiotic strains, with comparable methodology were recommended to reliably determine efficacy.^14^ These meta-analyses have underlined the high degree of heterogeneity not only between the design and methodology, but also in the IBS subtypes. Therefore, standardized trials, with validated tools, are needed to measure primary and secondary endpoints.

The overwhelming majority of biotic clinical studies in IBS have been performed with probiotics. However, many studies hypothesize that some of the mechanisms behind the probiotic health benefits could be independent of the viability of the cell and could instead be mediated by heat inactivated strains, or postbiotics. Postbiotics are defined as inanimate microorganisms and/or their components, which confer health benefits to the host.^9^ In this RCT, we refer to heat-treated strains as postbiotics. Postbiotics have been shown to have some advantages over live probiotics in terms of stability, safety, longer shelf-life and better standardization.^15^ Results from the first randomized, placebo-controlled clinical trial of the postbiotic *Bifidobacterium bifidum* MIMBb75 demonstrated alleviation of IBS symptoms in a real-life setting and the trial achieved its composite primary endpoint.^16^ However, RCTs on postbiotics and IBS are limited, and further research is needed in this space.

The aim of this study was to determine the efficacy of the probiotic *Bifidobacterium longum* (ES1) and the postbiotic heat-treated *Bifidobacterium longum* ES1 (HT-ES1) in improving gastrointestinal symptoms of participants with IBS-D. The primary outcome measure was the change in IBS-Symptom Severity Scale (IBS-SSS) scores and the secondary outcome measures were changes in QoL score, Abdominal Pain Numeric Rating Scale (APS‐NRS) score, The State-Trait Anxiety Inventory-Adults (STAI-AD) (-S and -T) score, stool consistency and safety parameters.

## 2. Materials and methods

### 2.1. Compliance with ethical standards

Study conducted in compliance with the Declaration of Helsinki and National Ethical Guidelines for Biomedical and Health Research involving Human Participants. The study was registered with the NIH ClinicalTrials.gov (Identifier: NCT05339243) and Clinical Trials Registry India (Registration Number: CTRI/2022/04/041875). Written informed consent was obtained from all participants prior to initiation of study procedures. Participants were not involved in the trial design, recruitment, or dissemination. We will consider public involvement, if possible, in upcoming trials.

### 2.2. Intervention

This 3-arm study involved oral administration of two capsules once daily for 84 days (12 weeks). These two capsule interventions consisted of either *Bifidobacterium longum* CECT 7347 (ES1) (1×10^9^ colony-forming units (CFU)/day); or heat-treated *Bifidobacterium longum* CECT 7347 (HT-ES1) (2.5×10^9^ cells/day equivalent to 50 mg/day when prepared from a postbiotic batch at a concentration of 5 × 10^10^ cells/g); or maltodextrin 250 mg as a control. The postbiotic is prepared through a proprietary method of heat-treatment of the probiotic solution followed by a drying step, to create a solid powder. Capsules were produced by ADM at their manufacturing facility in Somerset, United Kingdom.

All three investigational products were visually identical upon inspection, displaying the same size, color, and weight. Identical capsules were packed in matching bottles and labeled in accordance with the blinding protocol.

### 2.3. Study participants

This trial was conducted in India between April 2022 and January 2023. A total of 200 healthy participants meeting the following inclusion criteria were enrolled; male or female aged ≥18 to ≤65 years, diagnosed with IBS-D as per ROME IV criteria and an IBS-SSS ≥175. Full details of the inclusion and exclusion criteria are available in Supplementary Methods (Section 1).

### 2.4. Study design

**Figure 1.**
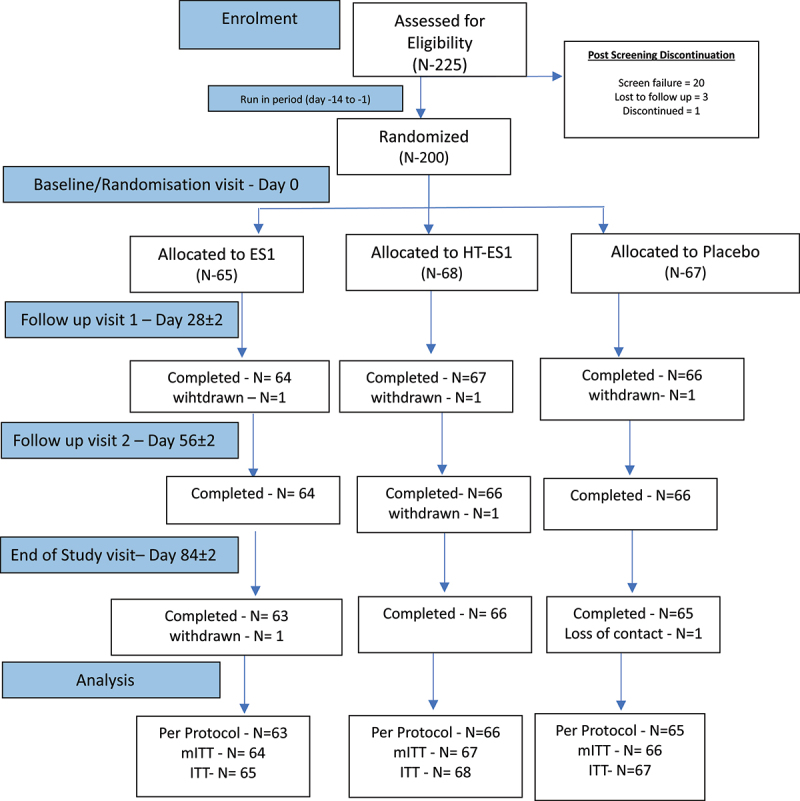
A CONSORT flow diagram illustrating the progress through the phases of the clinical trial.

A randomized, double-blind, placebo-controlled, parallel-group, multicentre, three arms study: Group 1: ES1, Group 2: HT-ES1 and Group 3: placebo. Participants were randomized in a 1:1:1 ratio according to a computer-generated blocked randomization list with a block size of six using StatsDirect Statistical Software version 3.1.17. Allocation was performed using CRFWEB by Clindox and concealed. A CONSORT flow diagram (Figure 1) and study visit schedule (Supplementary Methods (Section 2)) provide further details of the study design.

N = number of participants. In the ES1 arm two participants were withdrawn for protocol violation. In the HT-ES1 arm, one participant was withdrawn for low compliance and one for protocol violation. In the placebo arm, one was withdrawn because of an error in randomization and one loss of contact.

### 2.5. Safety and efficacy outcomes

#### 2.5.1. Safety variables

Blood pressure, pulse rate, laboratory parameters related to liver and kidney function (ALT, AST, ALP and creatinine) and adverse events (AEs)/serious adverse events (SAEs) were recorded throughout the study at the timepoints detailed in Supplementary Methods (Section 2).

#### 2.5.2. Primary outcomes

##### 2.5.2.1. IBS-SSS

The IBS-SSS is an internationally validated assessment tool used to evaluate the intensity of IBS symptoms.^17^ IBS-SSS is a composite score of abdominal pain, number of days with abdominal pain, bloating/distension, and satisfaction with bowel habits. Each measure is rated from 0 to 100, with total scores ranging from 0 to 500: scores between 75–175 are considered mild, 175–300 moderate or >300 severe IBS (Supplementary Methods Section 3).

#### 2.5.3. Secondary outcomes

##### 2.5.3.1. Responder analysis

A responder analysis was performed for this study, with results including the percentage of responders in each group. IBS-SSS is a validated tool with standard criteria for interpretation of symptoms severity, however there is no gold standard for interpretation of changes in the score. Three different approaches have been published in the past: IBS-SSS responders defined as participants who had a clinically significant change of either 50 points or 95 points^18^ in IBS-SSS total score from baseline or a 30% change in abdominal pain score from baseline.^19,20^ Evaluating study results against all three criteria allowed to better understand the magnitude of changes and relevance of the results, regardless of which criterion was to be applied.

##### 2.5.3.2. Stool consistency

The Bristol Stool Form Scale (BSFS) was used to categorize participants’ stools into stool types ranging from type 1 (hard lumps) to type 7 (watery diarrhea). Bristol stool form types 6 & 7 are categorized as loose or watery stools and type 3, 4 & 5 are considered normal stool type. BSFS was recorded at pre-determined time points over the course of the study (Supplementary Methods Section 2).

##### 2.5.3.3. Quality of life

The IBS-QoL is a 34‐item self-report quality-of-life questionnaire assessing the impact of IBS and its treatment. It contains eight disease-relevant domains: dysphoria, interference with activity, body image, health worry, food avoidance, social reaction, and sexual and relationship issues with each item rated on a 5‐point scale (34–170). For ease of comparison, the summed scores of the IBS-QoL were transformed to a 0–100 scale ranging from 0 (poor quality of life) to 100 (maximum quality of life).^21^ The minimal clinically important difference (MCID) on the IBS-QOL is defined as a change of 10 points in the total score.^22^

##### 2.5.3.4. Abdominal pain severity

The 11‐point Abdominal Pain Numeric Rating Scale (APS‐NRS) was used to assess participant abdominal pain, with 10 representing the most severe pain and 0 representing no pain. The recall period for APS-NRS is 7 days and the scores on days 28. 56 and 84 are presented as a mean of the four weekly scores prior to the study vist. The MCID on the APS-NRS is a change of 2.2 points in the total score.^18^

##### 2.5.3.5. Mental health

The State-Trait Anxiety Inventory-Adults (STAI-AD) is a 40 item self-report questionnaire used to assess anxiety. There are two subscales: the State Anxiety Scale (STAI-S) (20 items) evaluates the current state of anxiety; and the Trait Anxiety Scale (STAI-T) (20 items) evaluates “anxiety proneness”. The range of scores for each subtest is 20–80, with a higher score indicating greater anxiety.

Participants provided answers to their questionnaires using an app on their mobile phone. Each participant received account details that were protected by login ID and password which was automatically generated only for a participant’s personal registered mobile number.

### 2.6. Statistical analysis

Measurement data (continuous variables) were subjected to normality testing using the Shapiro–Wilk test. All normal data was analyzed using parametric tests, and non-normal data was analyzed using non-parametric tests for hypothesis testing. For continuous data, ANOVA and chi-square/fisher’s exact test for categorical variables were used to compare the baseline demographic data between groups. Change in IBS-SSS total score at the end of study and all other visits from baseline compared between the three groups were analyzed using analysis of covariance (ANCOVA). Dunnett’s post hoc test was used to compare investigational groups compared to the control arm to determine which investigational groups were statistically significantly different from the placebo. Within group comparison from baseline to post baseline assessment were done using paired t-test. Further information is provided in the Supplementary Methods (Section 3). All statistical analyses were performed on the modified intention to treat population (mITT), which comprised all randomized patients who received at least one dose of the interventional product [*n* = 64 (ES1), 67 (HT-ES1) and 66 (Placebo)].

#### 2.6.1. Determination of sample size

For pilot clinical trials assessing feasibility, a sample size between 24 and 50 has been recommended.^23–25^ In this trial, a total of 225 participants were screened. Of those, 200 participants who met the inclusion criteria were randomized with an anticipated dropout rate of 25% due to COVID-19 pandemic. Our aim was to have 150 completers at the end of the trial. The participants were randomized into three groups in the ratio of 1:1:1.

## 3. Results

### 3.1. Baseline characteristics of study participants

The three intervention groups were comparable with respect to the baseline demographic characteristics (Table 1). Further baseline parameters and concurrent medication use are detailed in Table S1 and S2. Of the 200 randomized participants, 197 completed the study to at least one follow-up visit (64, 67 and 66 participants in the ES1, HT-ES1 and placebo groups, respectively) and 194 participants completed the study until the end (63, 66 and 65 participants in the ES1, HT-ES1 and placebo groups, respectively).

**Table 1.** Participant demographics.

Parameter	Categories	ES1 (*N* = 65)	HT-ES1 (*N* = 68)	Placebo (*N* = 67)	Total (*N* = 200)
Age (years)	Mean (SD)	37.28 (11.04)	34.88 (9.56)	35.73 (11.42)	35.95 (10.68)
	Range (Min., Max)	(19.00, 58.00)	(20.00, 58.00)	(20.00, 63.00)	(19.00, 63.00)
Gender	Female	17 (26.15%)	30 (44.12%)	26 (38.81%)	73 (36.50%)
	Male	48 (73.85%)	38 (55.88%)	41 (61.19%)	127 (63.50%)
BMI (kg/m^2^)	Mean (SD)	23.26 (2.86)	22.62 (2.57)	22.29 (2.83)	22.72 (2.77)
	Range (Min., Max)	(17.04, 29.74)	(16.14, 29.28)	(16.91, 28.13)	(16.14, 29.74)

*BMI, body mass index; ES1, Bifidobacterium longum (group 1); HT-ES1, Heat-treated Bifidobacterium longum (group 2); kg/m*^*2*^, *kilogram per square meter; Max, maximum; Min, minimum; N, number of participants; SD, standard deviation.*

### 3.2. IBS-SSS total score

**Figure 2.**
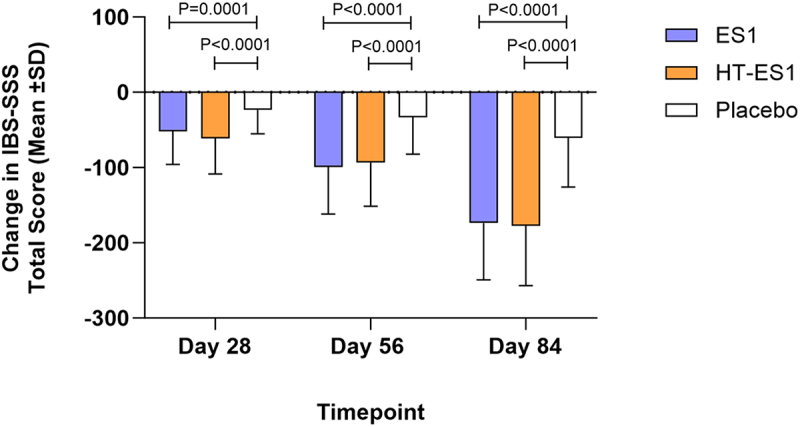
Change in mean IBS-SSS total score in the three groups at Day 28, 56 and 84.

At baseline, the mean IBS-SSS total score was comparable across the three intervention groups (Placebo: 299.38 [±47.05], ES1: 291.64 [±45.63] (*p* = .5219) and HT-ES1: 292.12 [±43.42] (*p* = .5550)) (Table 2). The change from baseline in IBS-SSS total score was statistically significant in the ES1 and HT-ES1 groups compared to the placebo group on Days 28, 56 and 84 (Table 2, Figure 2). The greatest mean change from baseline was observed at Day 84: (Placebo: −60.44 [±65.50] (*p* < .0001), ES1: −173.70 [±75.60] (*p* < .0001) and HT-ES1: −177.60 [±79.32] (*p* < .0001)) (Figure 2). This is equal to a 60.8% and 59.6% reduction in the IBS-SSS scores from baseline in the HT-ES1 and ES1 group respectively, compared to only a 21.2% reduction in the placebo group (*p* < .0001). The total IBS-SSS scores are presented in Figure S1. In both ES1 and HT-ES1 groups the total IBS-SSS scores were significantly lower compared to placebo on Days 28, 56 and 84.

The mean change in IBS-SSS total score compared to baseline scores for each treatment group on Days 28, 56 and 84 are presented. Error bars represent the SD. p-values were calculated using paired t-test (T).

*ES1, Bifidobacterium longum; HT-ES1, Heat-treated Bifidobacterium longum; IBS-SSS, Irritable Bowel Syndrome-Symptom Severity Scale; SD, standard deviation.*

**Table 2.** Change in IBS-SSS total score.

Visit	Categories	ES1 (*N* = 64)	HT-ES1 (*N* = 67)	Placebo (*N* = 66)	# p-value vs. placebo	* p-value vs. baseline
Baseline	Mean (SD)	291.64 (45.63)	292.12 (43.42)	299.38 (47.05)	ES1 0.5219 (T) HT-ES1 0.5550 (T)	– –
Day 28	Mean (SD)	−51.77 (44.00)	−61.10 (47.39)	−23.45 (31.61)	ES1 0.0001 (T) HT-ES1 < 0.0001 (T)	ES1 < 0.0001 (T) HT-ES1 < 0.0001 (T) Placebo <0.0001 (T)
Day 56	Mean (SD)	−99.14 (62.80)	−93.42 (58.00)	−33.03 (49.04)	ES1 < 0.0001 (T) HT-ES1 < 0.0001 (T)	ES1 < 0.0001 (T) HT-ES1 < 0.0001 (T) Placebo <0.0001 (T)
Day 84	Mean (SD)	−173.70 (75.60)	−177.60 (79.32)	−60.44 (65.50)	ES1 < 0.0001 (T) HT-ES1 < 0.0001 (T)	ES1 < 0.0001 (T) HT-ES1 < 0.0001 (T) Placebo <0.0001 (T)

*Baseline values (measured on Day 0) are listed. Day 28, 56 and 84 show the change in IBS-SSS score from baseline*.

*p-values were calculated using paired t-test (T).

# *Difference Estimate and p-values were calculated using ANCOVA with treatment and visit as factor and baseline as covariate vs. Placebo (Dunnett’s adjustment)*.

*ES1, Bifidobacterium longum; HT-ES1, Heat-treated Bifidobacterium longum; IBS-SSS, Irritable Bowel Syndrome Symptom Severity Score; N, number of participants; SD, standard deviation; vs., verses*.

In terms of IBS severity category, at Day 84, 46.15% of participants in ES1 group and 35.29% in the HT-ES1 group improved from Moderate to Mild category. 12.31% of participants in ES1 and 19.12% in HT-ES1 group improved from severe IBS to symptom free at Day 84 compared to 0% in the placebo group. The placebo group had 31.34% of participants who saw no improvement and stayed in moderate IBS category after 84 days (Figure S2).

### 3.3. Responder analysis

**Figure 3.**
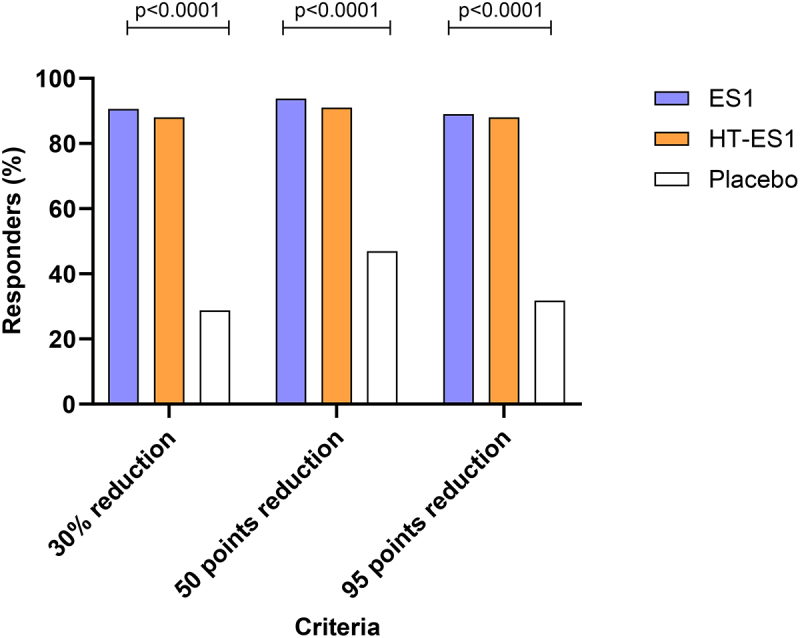
The percentage of responders for all three criteria on day 84.

The number of responders for all 3 analysis criteria was significantly higher in ES1 and HT-ES1 groups at all timepoints compared to the placebo group (Table 3). By Day 84, 90.63% of participants in the ES1 group and 88.06% in the HT-ES1 group reported a 30% reduction in abdominal pain from baseline scores as compared to only 28.79% in the placebo group. Similar results were observed for 95-point reduction in IBS-SSS scores from baseline, whereby the percentage of responders in the ES1 group was 89.06%, HT-ES1 group 88.06% and placebo group 31.82%. At Day 84 for 50-point reduction in IBS-SSS scores from baseline, the percentage of responders in the ES1 group was 93.75%, HT-ES1 group 91.04%, and placebo group 46.97%. The results were statistically significant for ES1 and HT-ES1 compared to placebo for all three criteria (Table 3, Figure 3).

The percentage of responders for three criteria are displayed. Firstly, the percentage of responders with a 30% reduction in abdominal pain score between baseline and Day 84. Next, the percentage of responders with a 50-point and 95-point reduction in IBS-SSS scores between baseline and Day 84. *p* values were calculated using Chi Square test.

*ES1, Bifidobacterium longum; HT-ES1, Heat-treated Bifidobacterium longum; IBS-SSS, Irritable Bowel Syndrome-Symptom Severity Scale; N, number of participants.*

**Table 3.** Responder analysis from baseline until study completion.

Analysis criteria	Visit	Responders/Non-responders	ES1 (*N* = 64)	HT-ES1 (*N* = 67)	Placebo (*N* = 66)	p-value*
30% reductions in abdominalpain from baseline score	Day 28	Responders N (%)	15 (23.44)	14 (20.90)	2 (3.03)	.0022 (C)
		Non-responders N (%)	49 (76.56)	53 (79.10)	64 (96.97)	
	Day 56	Responders N (%)	35 (54.69)	37 (55.22)	8 (12.12)	<.0001 (C)
		Non-responders N (%)	29 (45.31)	30 (44.78)	58 (87.88)	
	Day 84	Responders N (%)	58 (90.63)	59 (88.06)	19 (28.79)	<.0001 (C)
		Non-responders N (%)	6 (9.38)	8 (11.94)	47 (71.21)	
50 points reductions in IBS-SSS score from baseline	Day 28	Responders N (%)	32 (50.00)	37 (55.22)	12 (18.18)	<.0001 (C)
		Non-responders N (%)	32 (50.00)	30 (44.78)	54 (81.82)	
	Day 56	Responders N (%)	52 (81.25)	52 (77.61)	23 (34.85)	<.0001 (C)
		Non-responders N (%)	12 (18.75)	15 (22.39)	43 (65.15)	
	Day 84	Responders N (%)	60 (93.75)	61 (91.04)	31 (46.97)	<.0001 (C)
		Non-responders N (%)	4 (6.25)	6 (8.96)	35 (53.03)	
95 points reductions in IBS-SSSscore from baseline	Day 28	Responders N (%)	11 (17.19)	14 (20.90)	1 (1.52)	.0022 (C)
		Non-responders N (%)	53 (82.81)	53 (79.10)	65 (98.48)	
	Day 56	Responders N (%)	32 (50.00)	31 (46.27)	5 (7.58)	<.0001 (C)
		Non-responders N (%)	32 (50.00)	36 (53.73)	61 (92.42)	
	Day 84	Responders N (%)	57 (89.06)	59 (88.06)	21 (31.82)	<.0001 (C)
		Non-responders N (%)	7 (10.94)	8 (11.94)	45 (68.18)	

Percentages (%) were calculated using representative column header count as denominator.

*P-values were calculated using Chi Square (C) test.

*ES1, Bifidobacterium longum (group 1); HT-ES1, Heat-treated Bifidobacterium longum (group 2)*, *IBS-SSS, Irritable Bowel Syndrome Symptom Severity Score; N, number of participants*.

### 3.4. Stool consistency

**Figure 4.**
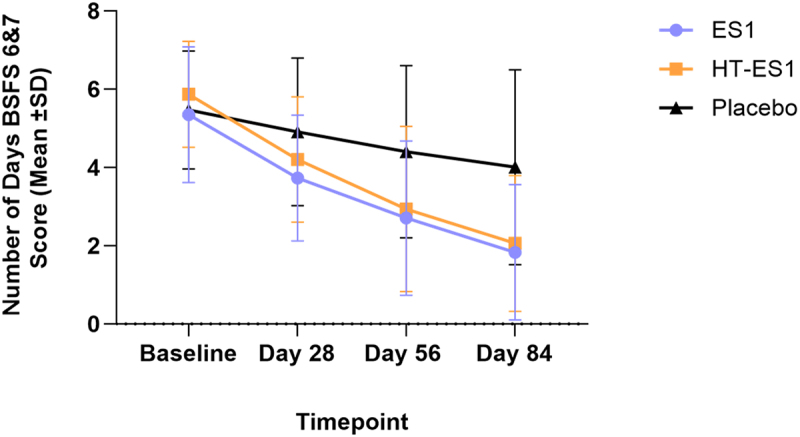
Mean number of days participants recorded BSFS types 6 & 7 scores.

The clinicaltrials.gov registration states that the outcome measure relating to stool consistency would be the percentage of the population achieving BSFS type 3,4 or 5. However, due to an oversight during case report form (CRF) development, the data collected cannot be expressed in this way. Instead, we present the mean number of days of BSFS stool types. After 84 days, the mean number of days per week with normal stool type was significantly higher in the ES1 and HT-ES1 groups compared to placebo [Placebo: 2.55 (±2.53), ES1: 4.63 (±1.92) (*p* < .0001), HT-ES1: 4.36 (±1.84) (*p* < .0001)]. By the end of the intervention period, there was a significant reduction in the average number of days participants experienced loose stool (BSFS type 6 & 7) in the ES1 and HT-ES1 groups compared to the placebo group (placebo: −1.46 [±1.99], ES1: −3.52 [±2.05] (*p* < .05), HT-ES: −3.81 [±2.00] (*p* < .05)) (Figure 4). The BSFS data recorded throughout the study can be found in Table S3.

The mean number of days of BSFS types 6 and 7 for ES1, HT-ES1 and placebo group at different timepoints are presented. Error bars represent the SD.

*BSFS, Bristol Stool Form Scale; ES1, Bifidobacterium longum; HT-ES1, Heat-treated Bifidobacterium longum; SD, standard deviation.*

### 3.5. Quality of life

**Figure 5.**
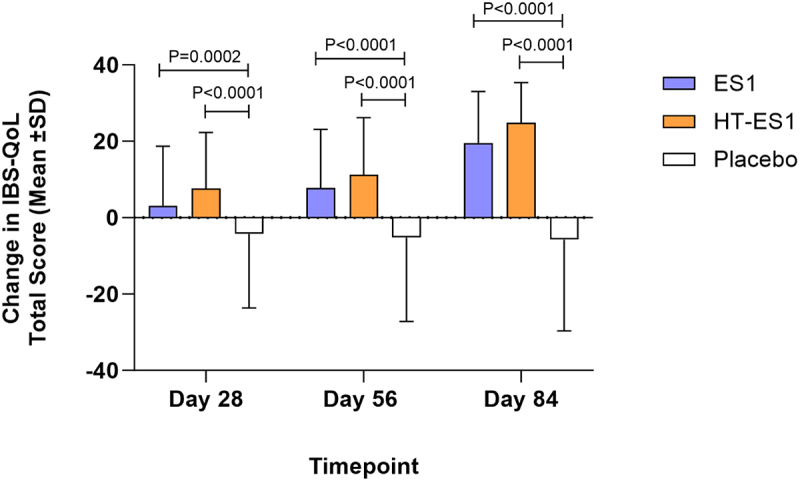
Change in the mean IBS-QoL total score in the three groups.

The mean IBS-QoL total score was comparable across the three study groups at baseline [Placebo: 62.46 (±18.34), ES1: 64.84 (±19.50) and HT-ES1: 59.99 (±20.23)] (Table S4). At Day 84, the change from baseline in mean IBS-QoL total score was significant in the ES1 and HT-ES1 study groups as compared to placebo group [Placebo: −5.74 (±16.07), ES1: 19.54 (±19.52) (*p* < .0001) and HT-ES1: 24.80 (±21.58) (*p* < .0001)] (Figure 5, Table S4). The improvement in IBS-QoL scores for ES1 and HT-ES1 compared to placebo was observed from as early as Day 28. The mean total IBS-QoL scores for all three groups are presented in Figure S3 and show significant improvement for all timepoints when compared to placebo group scores. The minimal clinically important difference (MCID) on the IBS-QoL is a change of 10 points in the total score and this study achieved > 10-point increase in QoL scores after 84 days of supplementation with ES1 and HT-ES1.

The mean change in IBS-QoL total score compared to baseline for each treatment group on Days 28, 56 and 84 is presented. Error bars represent the SD. p-values were calculated using paired t-test .

*ES1, Bifidobacterium longum; HT-ES1, Heat-treated Bifidobacterium longum; IBS-QoL, Irritable Bowel Syndrome-Quality of Life; SD, standard deviation.*

### 3.6. Abdominal pain severity

**Figure 6.**
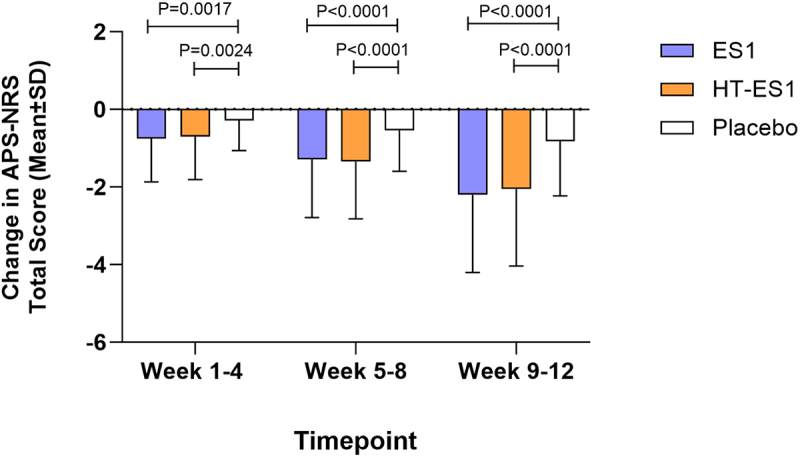
Change in mean APS-NRS total score in the three groups.

The mean APS-NRS scores at baseline were comparable between groups: [placebo: 6.48 (±0.86), ES1: 6.49 (±0.91) and HT-ES1: 6.36 (±1.13)]. The mean change in APS-NRS scores for each group from weeks 1–4 to weeks 9–12 is presented in Figure 6. The reduction in APS-NRS score by weeks 9–12 after ES1 and HT-ES1 supplementation was significant compared to placebo [placebo −0.88 (±1.32), ES1: −2.23 (±2.00) (*p* < .0001) and HT-ES1 -2.06 (±1.99) (*p* < .0001)]. Furthermore, the mean APS-NRS total score was reported to also be significantly different at weeks 1–4, 5–8 and 9–12 in the ES1 and HT-ES1 groups compared to the placebo group (Figure S4). The MCID was defined as a change of 2.2 points in the total score and based on this criterion, the percentage of responders for each group for weeks 9–12 was as follows: ES1: 53.13%, HT-ES1: 47.76%, and placebo 16.67% (*p* < .0001).

The mean change in APS-NRS total score compared to baseline for each treatment group between Weeks 1–4, 5–8 and 9–12 are presented. Error bars represent the SD. p-values were calculated using paired t-test.

*APS-NRS, Abdominal Pain Severity-Numeric Rating Scale; ES1, Bifidobacterium longum; HT-ES1, Heat-treated Bifidobacterium longum; SD, standard deviation.*

### 3.7. Mental health

**Figure 7.**
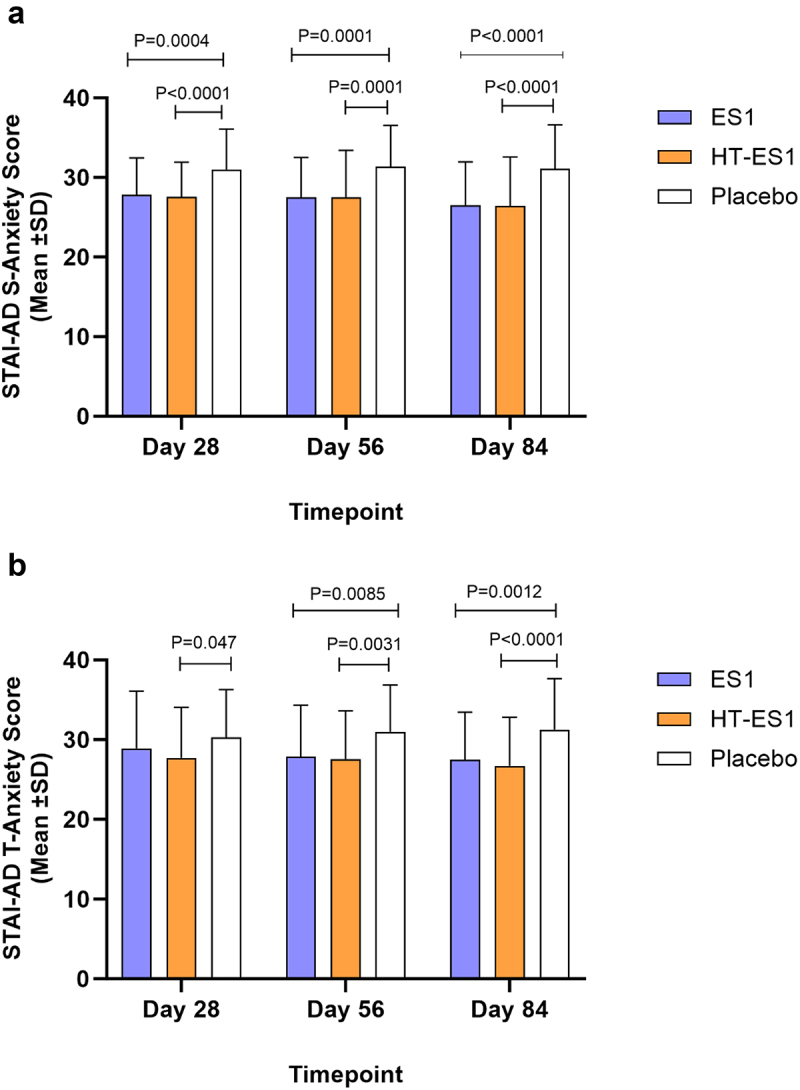
Mean STAI-AD score.

The mean STAI-AD scores (S-Anxiety and T-Anxiety) at baseline were comparable between groups, with no statistically significant differences. STAI S-anxiety baseline scores [placebo: +28.58 (±4.94), ES1: +28.66 (±4.43), HT-ES1: +29.04 (±4.50)] and T-anxiety baseline scores [placebo: +29.80 (±8.00), ES1: +29.06 (+5.53), HT-ES1: +29.93 (±7.38)]. Results showed a significant decrease of mean STAI-AD, S-Anxiety and T-anxiety scores after supplementation with ES1 and HT-ES1 compared to placebo control (Figure 7a, b). At Day 84, the changes in mean scores were: STAI-S [placebo: +2.53 (±6.01), ES1: −2.16 (±4.83) (*p* < .001), HT-ES1: −2.58 (±7.17) (*p* < .001)] and STAI-T [placebo: +1.44 (±5.70), ES1: −1.59 (±5.44) (*p* < .0002), HT-ES1: −3.21 (±6.19) (*p* < .0001)].

Mean reductions in S-Anxiety score (a). T-Anxiety scores (b) compared to baseline are presented. Error bars represent the SD and p-values were calculated using paired t-test .

*ES1, Bifidobacterium longum; HT-ES1, Heat-treated Bifidobacterium longum; SD, standard deviation; STAI-AD, State-Trait Anxiety Inventory-Adults.*

### 3.8. Rescue medication

Recuse medication was permitted to ensure participants safety and compliance, those who had over 5 bowel motions a day were allowed to use one dose of Loperamide for up to 3 days. Loperamide was used as a rescue medication amongst the participants in all groups throughout the study. There was a significant decrease in the average number of loperamide tablets in the ES1 and HT-ES1 groups at all time points compared to placebo (Table S5). At the end of study, between Days 57–84, average number of tablets used per participant was ES1: 2.19 (±2.93), HT-ES1: 2.43 (±3.06) vs Placebo: 5.58 (±5.22) (*p* < .0001).

### 3.9. Safety

All participants maintained stable levels of ALP, AST, ALT and creatinine throughout the study and there were no significant mean changes in vital signs (Table S6 and S7). In addition, across all treatment groups, AEs were mild and there were no SAEs reported (Table S8). A total of 22 participants across the study have reported AE. Of those, seven participants were in the HT-ES, nine in ES1 and six people in placebo arms.

## 4. Discussion

This study investigated the efficacy of the probiotic *Bifidobacterium longum* (ES1) and the postbiotic heat-treated *Bifidobacterium longum* ES1 (HT-ES1) in improving gastrointestinal symptoms of participants with IBS-D. The study met its primary endpoint and demonstrated that there was a significant decrease in IBS-SSS scores in both intervention groups compared to the placebo group, and the number of participants who reached the MCID for IBS-SSS scores was significantly higher in both intervention groups compared to the placebo group. Furthermore, all secondary outcomes reached statistical significance. The study also collected and analyzed fecal samples for metagenomic analysis, the results of which will be presented in a subsequent publication.

Similar results to those seen in this study were observed in a four-week study using the probiotic *Bifidobacterium infantis* 35624 in women with IBS.^26^ The positive effects of probiotic *Bifidobacterium infantis* 35624 were further confirmed in a subsequent, smaller, eight-week study of 77 patients with IBS.^27^ To date, there are only a small number of studies investigating the role of postbiotics of the *Bifidobacterium* genera in IBS, including an eight-week RCT conducted in 443 IBS patients. Results from this study demonstrated that heat-inactivated *Bifidobacterium bifidum* MIMBb75 substantially alleviated IBS symptoms.^16^ Collectively, these studies highlight the benefits of probiotic and postbiotic *Bifidobacterium* species in IBS; however, none of these studies investigated the effect on specific IBS subtypes. Whilst there have been several RCTs focussing on the use of multi-strain probiotics on IBS-D,^28–30^ this study is the first to specifically focus on a single strain probiotic and postbiotic in IBS-D. Results of the primary endpoint demonstrate a significant reduction of over 170 points in IBS-SSS scores in both intervention groups (ES1 and HT-ES1) after 84 days of supplementation, compared to a reduction of 60.44 points in the placebo group. Similar results to these were previously seen in an RCT using a multi-strain probiotic formulation (Bio-Kult®), with a 223-point reduction in IBS-SSS scores in the treatment group compared to baseline.^28^ However, the combination of large effect size and the novelty of having positive results from both a probiotic and postbiotic of the same strain, make this study highly significant.

Evaluation of the secondary endpoints further confirmed the positive effects of ES1 and HT-ES1. Stool consistency improved throughout the study, with participants in ES1 and HT-ES1 groups having on an average 5–6 days of loose stool at baseline, which decreased to an average of 1–2 days per week by Day 84. Similarly, the number of days per week with normal stool type, as defined by BSFS types 3, 4, 5, increased from 1.45 and 0.95 days at baseline to 4.63 and 4.36 days by the end of the study in the ES1 and HT-ES1 groups, respectively.

There was a significant improvement in IBS-QoL scores in the ES1 and HT-ES1 groups compared to the placebo group, including a significant improvement in QoL for participants with moderate to severe IBS. In addition, abdominal pain was significantly decreased at all timepoints in the ES1 and HT-ES1 groups compared to the placebo group. Mean STAI-AD score (S-Anxiety and T-Anxiety) also showed significant reductions in the ES1 and HT-ES1 groups compared to the placebo group, indicating a decrease in anxiety symptoms. However, these results should be viewed with the understanding that this population did not experience significant baseline levels of anxiety, so although statistically significant, the clinical relevance of these findings may be open to interpretation.

Finally, the incidence of AEs recorded in this study was low and there were no SAEs reported in any arms of the study. In addition, there were no significant abnormalities identified in any of the blood tests or vital signs. Considering these findings, supplementation with ES1 and HT-ES1 was found to be safe and well tolerated.

It should be noted that one previously published paper^31^ examined the effect of the live probiotic ES1 on serum zonulin and cytokine profiles in adults with IBS-D. This previous trial using ES1, while in a similar population, differs considerably from the main results presented here: the data here were generated using gold standard tools to assess IBS symptom severity, abdominal pain, quality of life and anxiety and also includes the postbiotic HT-ES1, which was absent from the work carried out by Caviglia and colleagues.

Noteworthy strengths of this study include the rigorous study design and duration of intervention/follow-up. Limitations/qualifications to note are firstly that although this was a multi-center RCT, all study sites were in the same country and IBS management practices and dietary habits may vary compared to other parts of the world. That being said, a recent review examining the epidemiology of IBS in India, Bangladesh and Malaysia observed that differences in prevalence rates of IBS in Eastern and Western populations might be attributed to differences in evaluation methods^32^. In our clinical trial, we used the ROME IV diagnostic criteria, which are consistent with clinical practice standards in Europe. This choice helped mitigate some potential sources of variability. Secondly, diet was not standardized during the trial and so it is possible that consumption of certain foods in the participants’ diet could have affected IBS symptoms, independent of intervention factors such as higher dietary fiber consumption, differing rates of lactose malabsorption, prevalence of parasitosis, post-infectious IBS, as well as the predominance of rural populations compared to Western IBS trials, could potentially have an impact that cannot be easily quantified in a study of this size. Thirdly, neither IBS-SSS nor IBS-QoL evaluates urgency specifically, no additional tool was used to focus on that aspect, although indirectly any impact on urgency would be measured by change in IBS-QoL scores where several questions link to urgency matters. Lastly, the mechanistic data collected in this trial are essentially limited to fecal metagenomic analyses, the results of which will be presented in a separate publication. In any future study, it will be essential to examine a broader array of mechanistic outcomes – such as the role of the immune system, especially relevant given the results here seen in the postbiotic group. However, to our knowledge, this is the first RCT to demonstrate that the same strain of *Bifidobacterium longum* in both live probiotic and heat-treated postbiotic form is effective in reducing symptom severity in adults with IBS-D, highlighting the potential for use of these pro- and post- biotics in this population.

While not designed as a mechanistic trial, studies such as this one may still aid in our understanding of the potential mechanisms of action. It is well documented that probiotics, and specifically *Bifidobacteria*, are known to help restore the gut microbiota, by competing with harmful bacteria and enhancing the growth of beneficial microbes.^33^

But a particularly interesting finding from this study is that both the probiotic and postbiotic groups reported a positive effect on anxiety levels. Therefore it is proposed that supplementation with this strain may have influenced the so-called *gut-brain axis*. There is a growing body of evidence demonstrating the impact of various probiotics on the modulation of neurotransmitters such as serotonin and gamma-aminobutyric acid (GABA).^34^ These compounds are involved in gut motility, sensation, and mood regulation, providing interesting future direction for reseach into the microbiome and IBS.^35^

## 5. Conclusion

Results demonstrated that ES1 and HT-ES1 were safe and well tolerated. The primary outcome was achieved and both ES1 and HT-ES1 were shown to be efficacious in reducing IBS symptom severity, as measured by the IBS-SSS. The study also met its secondary endpoints related to responder rates, stool consistency, QoL, abdominal pain severity and anxiety, when compared to placebo over the 84-day intervention period. Overall, these results demonstrate that both ES1 and HT-ES1 are strong candidates for further research into IBS, with future trials addressing some of the limitations of this study, including conducting RCTs in other geographical locations, controlling for dietary confounders and further exploring potential mechanisms of action.

## Supplementary Material

Supplemental Material

## Data Availability

The data that support the findings of this study are available from the corresponding author, RD, upon reasonable request.
