# Supplementary material for: A randomized double-blind, placebo-controlled trial to evaluate the safety and efficacy of live Bifidobacterium longum CECT 7347 (ES1) and heat-treated Bifidobacterium longum CECT 7347 (HT-ES1) in participants with diarrhea-predominant irritable bowel syndrome
Source: Gut Microbes. 2024 Apr 17;16(1):2338322. doi: 10.1080/19490976.2024.2338322 (PMC11028008; doi:10.1080/19490976.2024.2338322)
Supplement: Supplemental Material [file KGMI_A_2338322_SM2187.doc]

# **Supplementary Results**

## **Table S1: Baseline Parameters**

| **Parameter** | **Categories** | **ES1**  **(N=65)** | **HT-ES1**  **(N=68)** | **Placebo**  **(N=67)** | **Total**  **(N=200)** |
| --- | --- | --- | --- | --- | --- |
| **Predominant stool type based on last 3 months’ experience** | TYPE 6 | 42 (64.62%) | 45 (66.18%) | 43 (64.18%) | 130 (65.00%) |
|  | TYPE 7 | 23 (35.38%) | 23 (33.82%) | 24 (35.82%) | 70 (35.00%) |
|  | (> 4) | 13 (20.00%) | 14 (20.59%) | 11 (16.42%) | 38 (19.00%) |
| **Average frequency of abdominal pain per week (episodes/ week)** | (1 – 2) | 27 (41.54%) | 21 (30.88%) | 19 (28.36%) | 67 (33.50%) |
|  | (3 – 4) | 25 (38.46%) | 33 (48.53%) | 37 (55.22%) | 95 (47.50%) |
| **Is the abdominal pain related to defecation** | No | 7 (10.77%) | 6 (8.82%) | 5 (7.46%) | 18 (9.00%) |
|  | Yes | 58 (89.23%) | 62 (91.18%) | 62 (92.54%) | 182 (91.00%) |
| **Is the abdominal pain associated with a change in the frequency of stool** | No | 8 (12.31%) | 9 (13.24%) | 6 (8.96%) | 23 (11.50%) |
|  | Yes | 57 (87.69%) | 59 (86.76%) | 61 (91.04%) | 177 (88.50%) |
| **Is the abdominal pain associated with a change in the form (appearance) of stool** | No | 26 (40.00%) | 26 (38.24%) | 29 (43.28%) | 81 (40.50%) |
|  | Yes | 39 (60.00%) | 42 (61.76%) | 38 (56.72%) | 119 (59.50%) |
| **Physical activity assessment (screening)** | Moderately active | 41 (63.08%) | 42 (61.76%) | 43 (64.18%) | 126 (63.00%) |
|  | Seldom active or sedentary | 23 (35.38%) | 20 (29.41%) | 21 (31.34%) | 64 (32.00%) |
|  | Vigorously active | 1 (1.54%) | 6 (8.82%) | 3 (4.48%) | 10 (5.00%) |
| **Physical activity assessment (baseline)** | Moderately active | 40 (61.54%) | 42 (61.76%) | 45 (67.16%) | 127 (63.50%) |
|  | Seldom active or sedentary | 25 (38.46%) | 22 (32.35%) | 21 (31.34%) | 68 (34.00%) |
|  | Vigorously active | 0 (0.00%) | 4 (5.88%) | 1 (1.49%) | 5 (2.50%) |
| **Blood glucose (mg/dL) (screening)** | Fasting | 2 (3.08%) | 1 (1.47%) | 2 (2.99%) | 5 (2.50%) |
|  | Random | 63 (96.92%) | 67 (98.53%) | 65 (97.01%) | 195 (97.50%) |
| **Pulse rate (bpm) (screening)** | Mean (SD) | 81.40 (7.27) | 80.22 (7.77) | 80.81 (7.51) | 80.80 (7.50) |
|  |  |  |  |  |  |
| **Systolic blood pressure (mmHg) (screening)** | Mean (SD) | 116.82 (10.64) | 115.04 (10.32) | 117.07 (11.26) | 116.30 (10.73) |
| **Diastolic blood pressure (mmHg) (screening)** | Mean (SD) | 76.22 (5.30) | 75.44 (5.37) | 75.15 (5.76) | 75.60 (5.47) |
| **SpO_2_ (%) (screening)** | Mean (SD) | 97.78 (0.96) | 97.91 (0.89) | 97.96 (0.86) | 97.89 (0.90) |
| **Body temperature (°C) (baseline)** | Mean (SD) | 36.55 (0.30) | 36.55 (0.42) | 36.54 (0.41) | 36.55 (0.38) |
| **Pulse rate (bpm) (baseline)** | Mean (SD) | 80.26 (6.24) | 79.34 (6.65) | 80.22 (6.89) | 79.94 (6.58) |
| **Systolic blood pressure (mmHg) (baseline)** | Mean (SD) | 117.63 (9.87) | 115.85 (9.20) | 117.61 (10.03) | 117.02 (9.69) |
| **Diastolic blood pressure (mmHg) (baseline)** | Mean (SD) | 75.94 (5.66) | 76.59 (5.82) | 76.21 (6.07) | 76.25 (5.83) |
| **SpO_2_ (%) (baseline)** | Mean (SD) | 97.91 (0.86) | 97.99 (0.86) | 97.97 (0.87) | 97.96 (0.86) |
| **Serum ALP (U/L) (baseline)** | Mean (SD) | 80.06 (17.87) | 82.73 (20.16) | 83.24 (20.80) | 82.03 (19.62) |
| **Serum AST (U/L) (baseline)** | Mean (SD) | 24.62 (6.10) | 24.20 (6.75) | 25.02 (8.13) | 24.61 (7.02) |
| **Serum ALT (U/L) (baseline)** | Mean (SD) | 23.66  (9.70) | 22.63 (11.73) | 24.24 (10.62) | 23.50 (10.70) |
| **Serum creatinine (mg/dL) (baseline)** | Mean (SD) | 0.82 (0.15) | 0.76 (0.14) | 0.77 (0.13) | 0.78 (0.14) |
| **TSH (microIU/L) (screening)** | Mean (SD) | 2.13 (0.98) | 1.88 (0.83) | 1.90 (0.96) | 1.97 (0.93) |
| **Blood glucose (mg/dL) (screening)*** | Mean (SD) | 96.88 (14.94) | 97.47 (17.39) | 97.68 (15.71) | 97.35 (15.99) |

**Value based on a combination of fasting and random blood glucose sample values*

*ALP, alkaline phosphatase; ALT, alanine aminotransferase;* *AST, aspartate aminotransferase; bpm, beats per minute; °C, degree Celsius; ES1, Bifidobacterium longum; HT-ES1, Heat-treated Bifidobacterium longum; Max, maximum; mg/dL, milligrams per decilitre; microIU/L, micro-international units per milliliter; Min, minimum; mmHg, millimetre of mercury; N, number of participants; SpO_2_, oxygen saturation; SD, standard deviation; TSH, thyroid stimulating hormone; U/L, units per litre.*

## **Table S2: Contaminant Medication**

| **Medication** | **ES1**  **(N=65)** | **HT-ES1**  **(N=68)** | **Placebo**  **(N=67)** | **Total**  **(N=200)** |
| --- | --- | --- | --- | --- |
|  | **Number of participants (%)*** | | | |
| **Ambroxol + Salbutamol** | 0 (0) | 1 (1.47) | 0 (0) | 1 (0.50) |
| **Cetirizine + Paracetamol + Phenylephrine** | 0 (0) | 1 (1.47) | 0 (0) | 1 (0.50) |
| **Paracetamol** | 0 (0) | 0 (0) | 2 (2.99) | 2 (1.00) |

**Percentages were calculated using respective column header count as denominator*

*ES1, Bifidobacterium longum; HT-ES1, Heat-treated Bifidobacterium longum; N, number of participants.*

## **Table S3: Frequency of Predominant Stool Type**

| **Parameters** | **Visit** | **Categories** | **ES1**  (N=64) | **HT-ES1**  (N=67) | **Placebo**  (N=66) | ***p-value compared to placebo (T)** |
| --- | --- | --- | --- | --- | --- | --- |
| **Number of days - BSFS 6 and 7** | Baseline  (Day 0) | Mean (SD) | 5.35 (1.73) | 5.87 (1.35) | 5.47 (1.51) | ES1 0.8700  HT-ES1 0.2263 |
|  | Day 28 | Mean (SD) | 3.73 (1.61) | 4.20 (1.60) | 4.91 (1.89) | ES1 0.0002  HT-ES1 0.0337 |
|  | Day 56 | Mean (SD) | 2.71 (1.97) | 2.94 (2.11) | 4.40 (2.20) | ES1<0.0001  HT-ES1 0.0002 |
|  | Day 84 | Mean (SD) | 1.83 (1.73) | 2.06 (1.74) | 4.01 (2.49) | ES1<0.0001  HT-ES1 <0.0001 |
| **Number of days – BSFS type 1 and 2** | Baseline  (Day 0) | Mean (SD) | 0.00 (0.00) | 0.00 (0.00) | 0.00 (0.00) | - |
|  | Day 28 | Mean (SD) | 0.02 (0.08) | 0.01 (0.04) | 0.01 (0.04) | ES1 0.6222  HT-ES1 0.9999 |
|  | Day 56 | Mean (SD) | 0.04 (0.15) | 0.05 (0.16) | 0.03 (0.14) | ES1 0.9988  HT-ES1 0.8075 |
|  | Day 84 | Mean (SD) | 0.11 (0.29) | 0.10 (0.32) | 0.11 (0.33) | ES1 0.9955  HT-ES1 0.9609 |
| **Number of days - BSFS type 3, 4 and 5** | Baseline  (Day 0) | Mean (SD) | 1.45 (1.77) | 0.95 (1.39) | 1.30 (1.59) | ES1 0.8119  HT-ES1 0.3315 |
|  | Day 28 | Mean (SD) | 2.93 (1.70) | 2.47 (1.67) | 1.86 (1.95) | ES1 0.0016  HT-ES1 0.0888 |
|  | Day 56 | Mean (SD) | 3.92 (2.00) | 3.71 (2.14) | 2.27 (2.20) | ES1 <0.0001  HT-ES1 0.0002 |
|  | Day 84 | Mean (SD) | 4.63 (1.92) | 4.36 (1.84) | 2.55 (2.53) | ES1 <0.0001  HT-ES1 <0.0001 |

**p-values were calculated using t-test (T) (Dunnett’s Adjustment)*

*BSFS, Bristol Stool Form Scale; ES1, Bifidobacterium longum; HT-ES1, Heat-treated Bifidobacterium longum; N, number of participants; NE, not equal; SD, standard deviation.*

## **Table S4: Change for Total IBS-QoL score**

| **Visit** | **Categories** | **ES1**  **(N=64)** | **HT-ES1**  **(N=67)** | **Placebo**  **(N=66)** | ***p-value vs. baseline (T)** | ***p-value vs. placebo**  **(T)** |
| --- | --- | --- | --- | --- | --- | --- |
| **Baseline** | Mean  (SD) | 64.84  (19.50) | 59.99  (20.23) | 62.46  (18.34) | ̶ | ES1 0.7051  HT-ES1 0.6833 |
| **Day 28** | Mean  (SD) | 3.18  (15.75) | 7.61  (14.11) | -4.32  (14.34) | ES1 0.1109  HT-ES1 <0.0001  Placebo 0.0170 | ES1 0.0002  HT-ES1 <0.0001 |
| **Day 56** | Mean  (SD) | 7.73  (19.00) | 11.24  (18.93) | -5.25  (14.18) | ES1 0.0018  HT-ES1 <0.0001  Placebo 0.0038 | ES1 <0.0001  HT-ES1 <0.0001 |
| **Day 84** | Mean  (SD) | 19.54  (19.52) | 24.80  (21.58) | -5.74  (16.07) | ES1 <0.0001  HT-ES1 <0.0001  Placebo 0.0051 | ES1 <0.0001  HT-ES1 <0.0001 |

*p-values were calculated using paired t-test (T).

*ES1, Bifidobacterium longum; HT-ES1, Heat-treated Bifidobacterium longum; N, number of participants; SD, standard deviation.*

## **Table S5: Summary of Rescue Medication (Number of Tablets)**

| **Visit** | **Number of Loperamide tablets** | **ES1**  **(N=64)** | **HT-ES1**  **(N=67)** | **Placebo**  **(N=66)** | ***P value compared to placebo** |
| --- | --- | --- | --- | --- | --- |
| **Run in period (Day -14 to -1)** | Mean (SD) | 2.92 (2.02) | 2.99 (2.11) | 3.09 (2.17) | 0.8978 |
| **Day 0 – 28** | Mean (SD) | 4.14 (3.92) | 4.12 (3.85) | 6.14 (5.43) | 0.0130 |
| **Day 29 – 56** | Mean (SD) | 3.58 (4.11) | 3.27 (3.69) | 5.70 (5.33) | 0.0033 |
| **Day 57 – 84** | Mean (SD) | 2.19 (2.93) | 2.43 (3.06) | 5.58 (5.22) | <0.0001 |

**p-values were calculated using ANOVA Test*

*ES1, Bifidobacterium longum; HT-ES1, Heat-treated Bifidobacterium longum; N, number of participants; SD, standard deviation.*

## **Table S6: Summary of Laboratory Parameters**

| **Parameter** | **Visit** | **Categories** | **ES1** | **HT-ES1** | **Placebo** | ***p-value compared to placebo (T)** |
| --- | --- | --- | --- | --- | --- | --- |
| **Serum ALP (U/L)** | **Baseline**  **(Day 0)** | N | 65 | 68 | 67 |  |
|  |  | Mean (SD) | 80.06 (17.87) | 82.73 (20.16) | 83.24 (20.80) | ES1 0.5507  HT-ES1 0.9838 |
|  | **Day 84** | N | 63 | 66 | 64 |  |
|  |  | Mean (SD) | 80.61 (17.69) | 83.25 (21.45) | 78.65 (19.15) | ES1 0.7947  HT-ES1 0.3031 |
| **Serum AST (U/L)** | **Baseline**  **(Day 0)** | N | 65 | 68 | 67 |  |
|  |  | Mean (SD) | 24.62 (6.10) | 24.20 (6.75) | 25.02 (8.13) | ES1 0.9228  HT-ES1 0.7192 |
|  | **Day 84** | N | 63 | 66 | 64 |  |
|  |  | Mean (SD) | 23.83 (5.05) | 23.60 (5.04) | 23.97 (7.22) | ES1 0.9859  HT-ES1 0.9071 |
| **Serum ALT (U/L)** | **Baseline**  **(Day 0)** | N | 65 | 68 | 67 |  |
|  |  | Mean (SD) | 23.66 (9.70) | 22.63 (11.73) | 24.24 (10.62) | ES1 0.9320  HT-ES1 0.5892 |
|  | **Day 84** | n | 63 | 66 | 64 |  |
|  |  | Mean (SD) | 23.01 (7.73) | 22.52 (7.41) | 22.74 (10.71) | ES1 0.9788  HT-ES1 0.9839 |
| **Serum creatinine (mg/dL)** | **Baseline**  **(Day 0)** | N | 65 | 68 | 67 |  |
|  |  | Mean (SD) | 0.82 (0.15) | 0.76 (0.14) | 0.77 (0.13) | ES1 0.0714  HT-ES1 0.8801 |
|  | **Day 84** | N | 63 | 66 | 64 |  |
|  |  | Mean (SD) | 0.78  (0.13) | 0.72  (0.16) | 0.75  (0.15) | ES1 0.3997  HT-ES1 0.3945 |

**p-values were calculated using t-test (T) (Dunnett’s Adjustment)*

*ALP, alkaline phosphatase; ALT, alanine aminotransferase;* *AST, aspartate aminotransferase; mg/dL, milligrams per decilitre; N, number of participants; SD, standard deviation; U/L, units per litre.*

## **Table S7: Summary of Vital Parameters**

| **Parameter** | **Visit** | **Categories** | **ES1** | **HT-ES1** | **Placebo** |
| --- | --- | --- | --- | --- | --- |
| **Pulse rate (bpm)** | **Baseline (Day 0)** | N | 65 | 68 | 67 |
|  |  | Mean (SD) | 80.26 (6.24) | 79.34 (6.65) | 80.22 (6.89) |
|  | **Day 28** | N | 64 | 67 | 66 |
|  |  | Mean (SD) | 80.22 (5.37) | 80.46 (6.17) | 79.74 (6.14) |
|  | **Day 56** | N | 64 | 66 | 66 |
|  |  | Mean (SD) | 79.23 (5.82) | 79.08 (6.06) | 79.47 (5.37) |
|  | **Day 84** | N | 63 | 66 | 65 |
|  |  | Mean (SD) | 79.73 (5.33) | 79.09 (5.50) | 79.05 (4.92) |
| **Systolic blood pressure (mmHg**) | **Baseline (Day 0)** | N | 65 | 68 | 67 |
|  |  | Mean (SD) | 117.63 (9.87) | 115.85 (9.20) | 117.61 (10.03) |
|  | **Day 28** | N | 64 | 67 | 66 |
|  |  | Mean (SD) | 118.03 (9.64) | 116.00 (8.94) | 117.50 (9.41) |
|  | **Day 56** | N | 64 | 66 | 66 |
|  |  | Mean (SD) | 117.19 (10.06) | 115.48 (9.00) | 117.12 (9.40) |
|  | **Day 84** | N | 63 | 66 | 65 |
|  |  | Mean (SD) | 116.90 (10.14) | 116.21 (8.30) | 116.54 (8.36) |
| **Diastolic blood pressure (mmHg)** | **Baseline (Day 0)** | N | 65 | 68 | 67 |
|  |  | Mean (SD) | 75.94 (5.66) | 76.59 (5.82) | 76.21 (6.07) |
|  | **Day 28** | N | 64 | 67 | 66 |
|  |  | Mean (SD) | 76.50 (5.57) | 77.42 (6.18) | 75.26 (5.95) |
|  | **Day 56** | N | 64 | 66 | 66 |
|  |  | Mean (SD) | 76.88 (4.80) | 75.56 (5.38) | 75.58 (5.68) |
|  | **Day 84** | N | 63 | 66 | 65 |
|  |  | Mean (SD) | 76.05 (5.64) | 77.48 (5.81) | 75.58 (5.50) |
| **SpO_2_ (%)** | **Baseline (Day 0)** | N | 65 | 68 | 67 |
|  |  | Mean (SD) | 97.91 (0.86) | 97.99 (0.86) | 97.97 (0.87) |
|  | **Day 28** | N | 64 | 67 | 66 |
|  |  | Mean (SD) | 98.00 (0.87) | 98.00 (0.72) | 97.97 (0.80) |
|  | **Day 56** | N | 64 | 66 | 66 |
|  |  | Mean (SD) | 98.13 (0.81) | 98.00 (0.94) | 98.26 (0.83) |
|  | **Day 84** | N | 63 | 66 | 65 |
|  |  | Mean (SD) | 98.16 (0.83) | 98.09 (0.76) | 98.17 (0.80) |
| **Body temperature (°C)** | **Baseline (Day 0)** | N | 65 | 68 | 67 |
|  |  | Mean (SD) | 36.55 (0.30) | 36.56 (0.41) | 36.52 (0.43) |
|  | **Day 28** | N | 64 | 67 | 66 |
|  |  | Mean (SD) | 36.55 (0.30) | 36.56 (0.33) | 36.52 (0.31) |
|  | **Day 56** | N | 64 | 66 | 66 |
|  |  | Mean (SD) | 36.52 (0.26) | 36.55 (0.29) | 36.56 (0.28) |
|  | **Day 84** | N | 63 | 66 | 65 |
|  |  | Mean (SD) | 36.58 (0.30) | 36.63 (0.30) | 36.58 (0.31) |

*bpm, beats per minute; °C, degree Celsius; mmHg, millimetres of mercury; N, number of participants; SD, standard deviation; SpO_2_, oxygen saturation.*

## **Table S8: Summary of Adverse Events**

| **Adverse event** | **ES1**  **(N=65)** | **HT-ES1**  **(N=68)** | **Placebo**  **(N=67)** | **Total**  **(N=200)** |
| --- | --- | --- | --- | --- |
|  | **(Number of events [%]*)** | | | |
| **Acidity** | 0 (0) | 0 (0) | 1 (1.49) | 1 (0.50) |
| **Body ache** | 0 (0) | 1 (1.47) | 1 (1.49) | 2 (1.00) |
| **Burning sensation in upper abdomen** | 1 (1.54) | 0 (0) | 0 (0) | 1 (0.50) |
| **Cold** | 0 (0) | 1 (1.47) | 0 (0) | 1 (0.50) |
| **Cough** | 0 (0) | 1 (1.47) | 0 (0) | 1 (0.50) |
| **Diarrhoea** | 0 (0) | 0 (0) | 1 (1.49) | 1 (0.50) |
| **Diastolic BP 58 (< normal range)** | 0 (0) | 1 (1.47) | 0 (0) | 1 (0.50) |
| **Fever** | 2 (3.08) | 0 (0) | 2 (2.99) | 4 (2.00) |
| **Hand Burn** | 1 (1.54) | 0 (0) | 0 (0) | 1 (0.50) |
| **Headache** | 4 (6.15) | 3 (4.41) | 1 (1.49) | 8 (4.00) |
| **Hoarseness** | 1 (1.54) | 0 (0) | 0 (0) | 1 (0.50) |
| **Knee pain** | 0 (0) | 1 (1.47) | 0 (0) | 1 (0.50) |
| **Muscular pain** | 0 (0) | 1 (1.47) | 0 (0) | 1 (0.50) |
| **Nausea and vomiting sensation** | 0 (0) | 0 (0) | 1 (1.49) | 1 (0.50) |
| **Skin rash** | 1 (1.54) | 0 (0) | 0 (0) | 1 (0.50) |
| **Rhinitis** | 1 (1.54) | 0 (0) | 0 (0) | 1 (0.50) |
| **Vomiting (1 episode)** | 0 (0) | 0 (0) | 1 (1.49) | 1 (0.50) |

**Percentages were calculated using respective column header count as denominator*

*BP, blood pressure; ES1, Bifidobacterium longum; HT-ES1, Heat-treated Bifidobacterium longum; N, number of participants.*

# **Supplementary Figures**


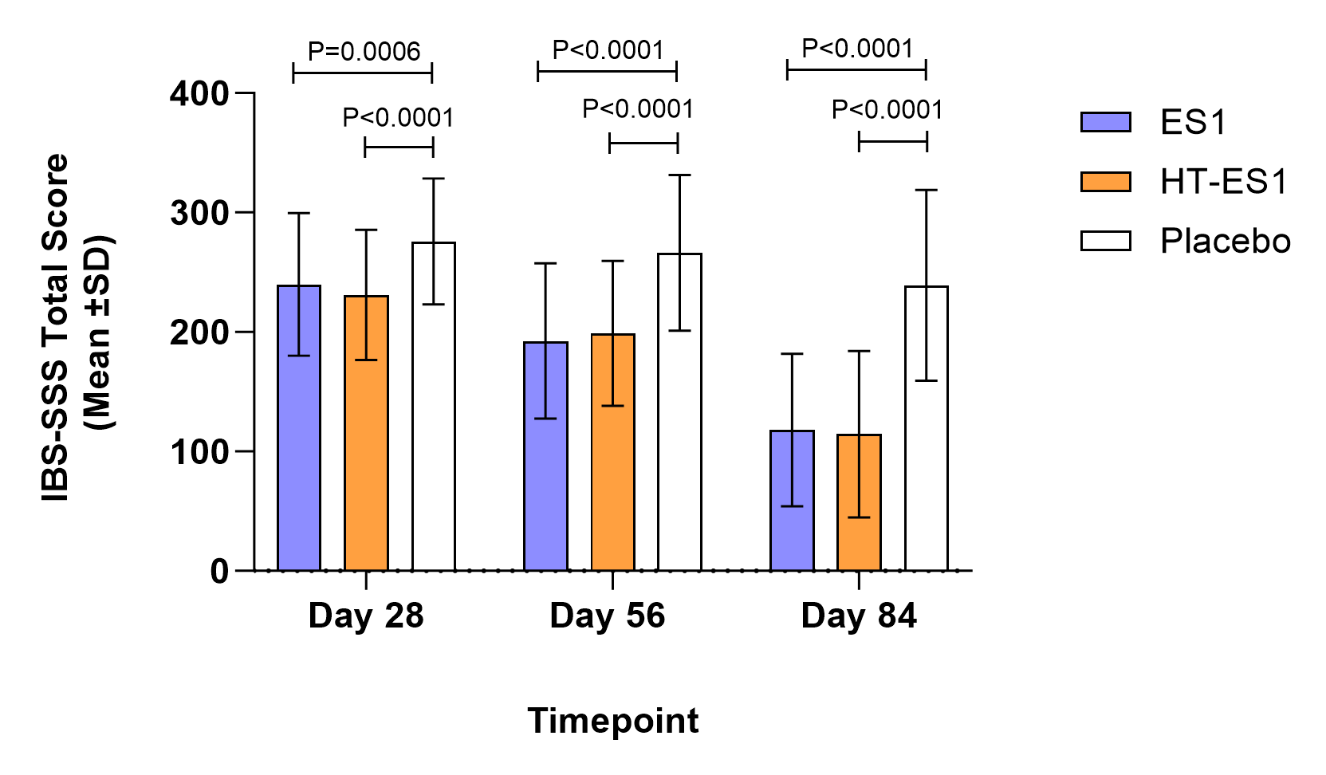


**Figure S1. Mean IBS-SSS total score in the three groups**. The mean IBS-SSS total scores at Days 28, 56 and 84 are presented. Error bars represent the SD. p-values were calculated using paired t-test (T).

*ES1, Bifidobacterium longum; HT-ES1, Heat-treated Bifidobacterium longum; IBS-SSS, Irritable Bowel Syndrome-Symptom Severity Scale; SD, standard deviation.*

*
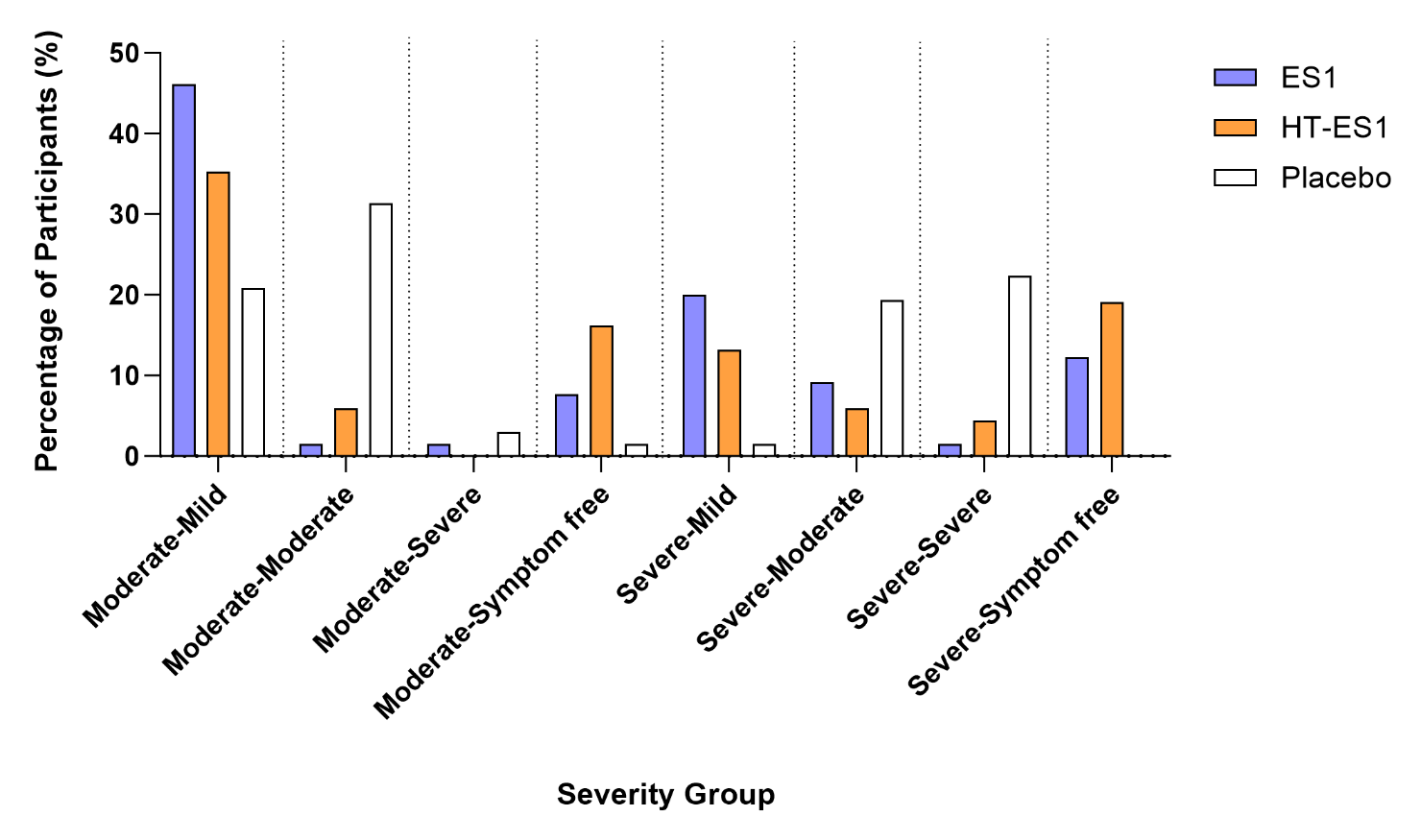
*

**Figure S2. Percentage of participants in each IBS-SSS severity group on Day 84**. The change in IBS severity group was assessed at baseline and Day 84 for all participants and the percentage of participants who’s severity category changed or remained the same between baseline and Day 84 are presented.

*ES1, Bifidobacterium longum; HT-ES1, Heat-treated Bifidobacterium longum; IBS-SSS, Irritable Bowel Syndrome-Symptom Severity Scale.*


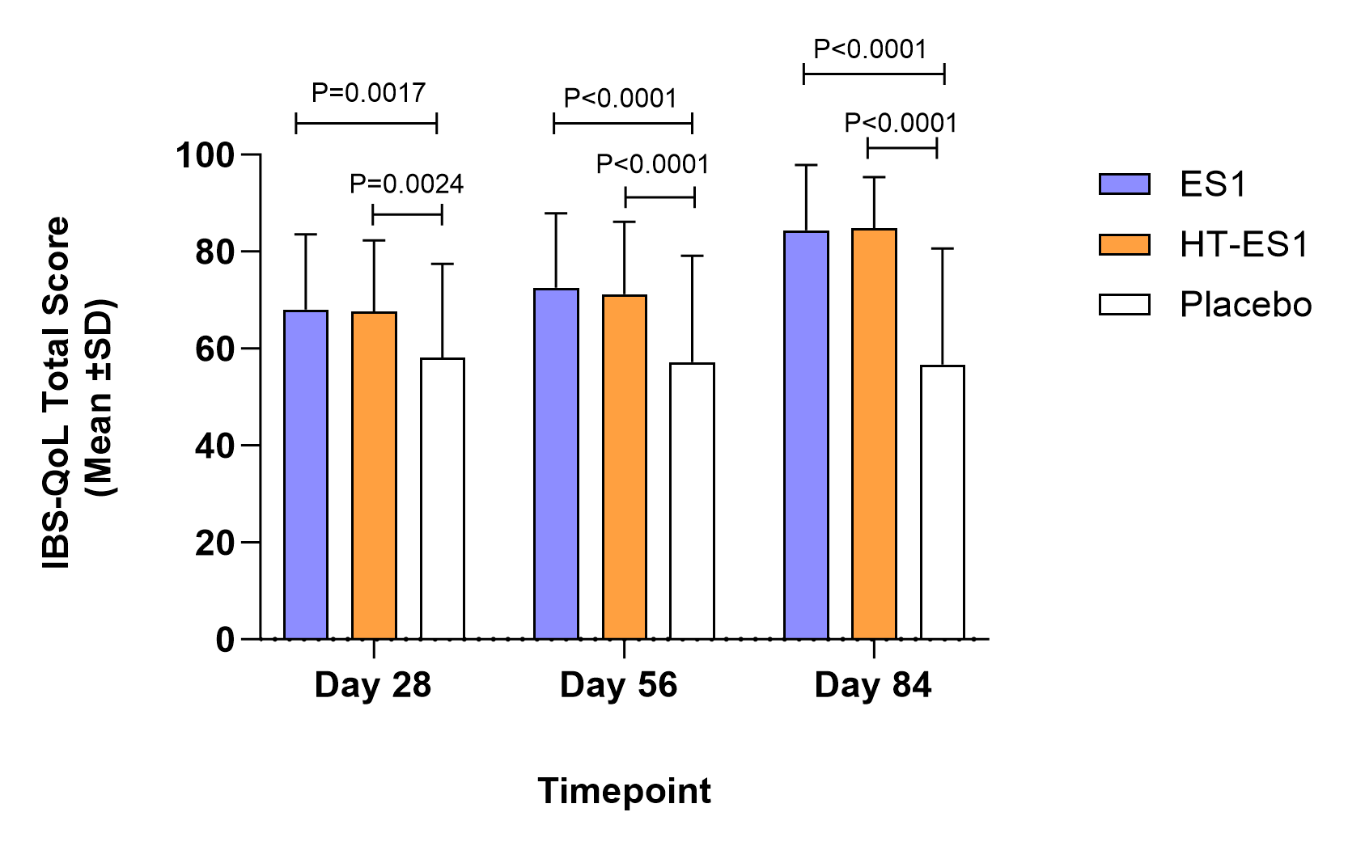


**Figure S3.** **Mean IBS-QoL total score in the three groups**. The mean IBS-QoL total scores at Days 28, 56 and 84 are presented. Error bars represent the SD. p-values were calculated using paired t-test (T).

*ES1, Bifidobacterium longum; HT-ES1, Heat-treated Bifidobacterium longum; IBS-QoL, Irritable Bowel Syndrome-Quality of Life; SD, standard deviation.*


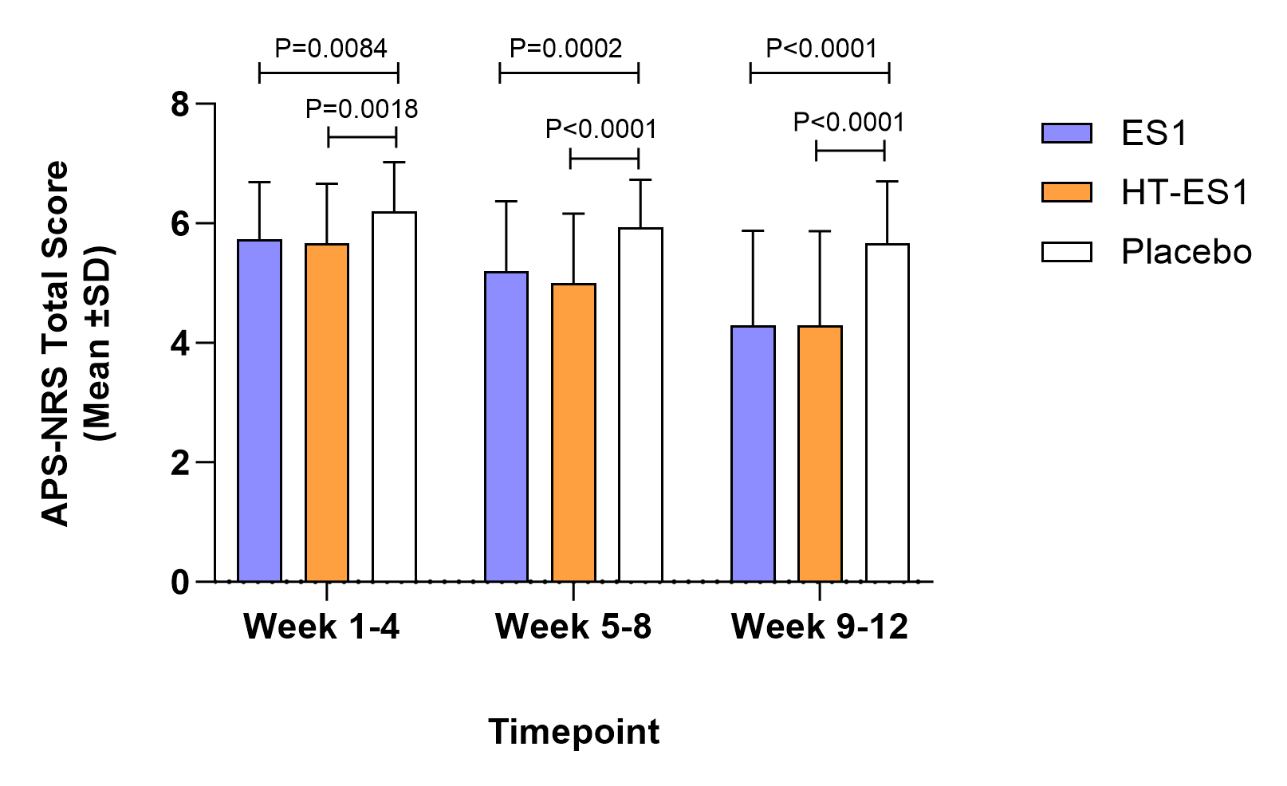


**Figure S4.** **Mean APS-NRS total score in the three groups.** The mean APS-NRS total score between Weeks 1 – 4, 5 – 8 and 9 – 12 are presented. Error bars represent the SD. p-values were calculated using paired t-test (T).

*APS-NRS, Abdominal Pain Severity-Numeric Rating Scale; ES1, Bifidobacterium longum; HT-ES1, Heat-treated Bifidobacterium longum; SD, standard deviation.*
